# A Novel Spatial Correlation Analysis of Peripheral Hemodynamics in a Murine Model of Vascular Calcification Using NIRS Imaging

**DOI:** 10.1007/s10439-026-04054-4

**Published:** 2026-03-24

**Authors:** Aasma Dahal, Shirel Belilty Benmergui, Daniela Leizaola, Valentina Dargam, Joshua Hutcheson, Anuradha Godavarty

**Affiliations:** 1 Optical Imaging Laboratory, Department of Biomedical Engineering, Florida International University, Miami, FL 33174 USA; 2 Cardiovascular Matrix Remodeling Laboratory, Department of Biomedical Engineering, Florida International University, Miami, FL 33174 USA; 3 Optical Imaging Laboratory, Department of Biomedical Engineering, Virginia Commonwealth University, Richmond, VA 23284 USA

**Keywords:** Near-infrared spectroscopy, Diffuse reflectance, Tissue oxygenation maps, Hemodynamic parameters, Vascular calcification, Chronic kidney disease

## Abstract

Vascular calcification (VC) is a major contributor to cardiovascular diseases (CVD) and progresses rapidly in patients with chronic kidney disease (CKD). Recently, we developed a low-cost, near-infrared spectroscopy (NIRS)-based imaging device (near-infrared optical scanner–NIROS) and used it to detect VC in the aortae via peripheral vascular imaging of the tail in mouse models. Our initial studies indicated distinct hemodynamic changes in the murine tail with and without the presence of VC. This study aims to assess the overall changes in peripheral hemodynamic flow patterns in mice with and without VC using NIROS. The tails of CKD mice with and without VC were imaged using NIROS to obtain the diffuse reflected signals, which were in turn used to calculate the spatiotemporal hemodynamic maps. Pearson’s correlation analysis was performed to obtain the flow correlation maps based on hemodynamic parameters and the NIR diffuse reflectance signals. Results demonstrated a significant decrease (*p*-value < 0.05) in hemodynamic flow correlation in the VC group as compared to those without VC. In addition, the flow correlations obtained from both NIRS wavelengths, 682 nm and 826 nm, showed a distinct decrease in correlation (*p*-value < 0.05), suggesting a disruption in the flow pattern once the mice developed VC. These results demonstrate the potential of peripheral flow correlation patterns using hemodynamic or NIRS diffuse reflectance signals as an indicator for aortic calcification detection. Future work will focus on correlating these peripheral hemodynamic changes, both peripheral and aortic VC, in a larger cohort of mice.

## Introduction

VC is a leading contributor to CVD and is associated with mortality and morbidity in patients with chronic kidney disease (CKD) [1]. Monitoring the degree and progression of VC, especially in CKD, where VC progresses rapidly, has a high prognostic value [2]. VC contributes to reduced peripheral blood perfusion, eventually leading to limb ischemia [3]. The decreased peripheral perfusion in patients with CKD and VC increases the risk of peripheral arterial disease (PAD), which, if not diagnosed and treated early, can progress to more severe conditions that require amputation [4]. Therefore, early detection and monitoring of VC are crucial for preventing and managing related complications. Despite significant research and development focusing on understanding the pathogenesis of VC, current techniques to assess existing VC require expensive imaging modalities that are not suitable for longitudinal monitoring. Existing modalities for diagnosing and monitoring VC, such as magnetic resonance imaging (MRI), computed tomography (CT), duplex ultrasonography, and echocardiography, have several limitations [5]. Although CT and MRI are sensitive to direct VC detection, their expense makes them unsuitable for regular monitoring of VC progression in CKD patients and limits accessibility to underserved populations [5]. Similarly, ultrasonography and echocardiography are used to visualize obvious large calcifications, but they have limited sensitivity to small or early vascular calcifications [6]. In addition, the ankle-brachial index (ABI), which is a widely used indirect method for detecting altered hemodynamics in the peripheries due to VC, suffers from accuracy and reliability issues, often requiring confirmation through other techniques [7]. Thus, there is a need for low-cost, accessible imaging techniques to detect the onset and progression of VC over time.

In our previous study, an in-house, low-cost, non-contact, and non-invasive spatiotemporal near-infrared spectroscopy (NIRS)-based imaging device (near-infrared optical scanner or NIROS) was used to detect changes in peripheral hemodynamics in the presence of CKD-induced VC [8]. This past study was conducted to identify the potential indicators for VC using peripheral hemodynamic parameters from discrete point locations from the murine tail in response to occlusion in the peripheries. The rate and extent of change in the hemodynamic parameters, i.e., oxy (∆HbO), de-oxy (∆HbR), total hemoglobin (∆HbT), and oxygen saturation (StO_2_), caused by vascular occlusion in the peripheries were measured [9]. The rate and extent of changes showed a significant decrease in the ∆HbO and ∆HbT; however, the ∆HbR and StO_2_ did not show significant differences between mice with CKD-induced VC compared to the CKD mice without VC. These observations were based on the hemodynamic changes at discrete point locations (located at the center of the tail’s cross-section and closer to the mouse’s body). Herein, the current study will focus on examining the overall hemodynamic flow pattern changes across the entire tail and determine if the spatiotemporal flow pattern changes could also serve as an indicator for detecting the presence of vascular calcification.

## Materials and Methods

### Animal Models

The results presented in this study are new analyses of data obtained from our previous study [8, 9]. A diet-based CKD mouse model (Adult C57BL/6J mice of 10 weeks of age) was used to obtain the occlusion-induced changes in peripheral hemodynamics. 10-week-old adult wild-type (C57BL/6J) mice were housed in the Animal Care Facility at Florida International University following an approved Institutional Animal Care and Use Committee (IACUC) protocol. These mice were kept on an adenine-enriched (0.2%) diet for 6 weeks to induce renal dysfunction leading to CKD. After 6 weeks on the diet, these mice were divided into two groups: the control group and the test group. The control group (CKD, *n* = 6 mice) continued on the same diet for an additional 6 weeks, while the test group (CKD+VC, *n* = 5 mice) was given an adenine-enriched diet with high phosphate (1.8%) to induce VC.

### Instrumentation and Image Acquisition

#### NIRS-Based Imaging

**Fig. 1:**
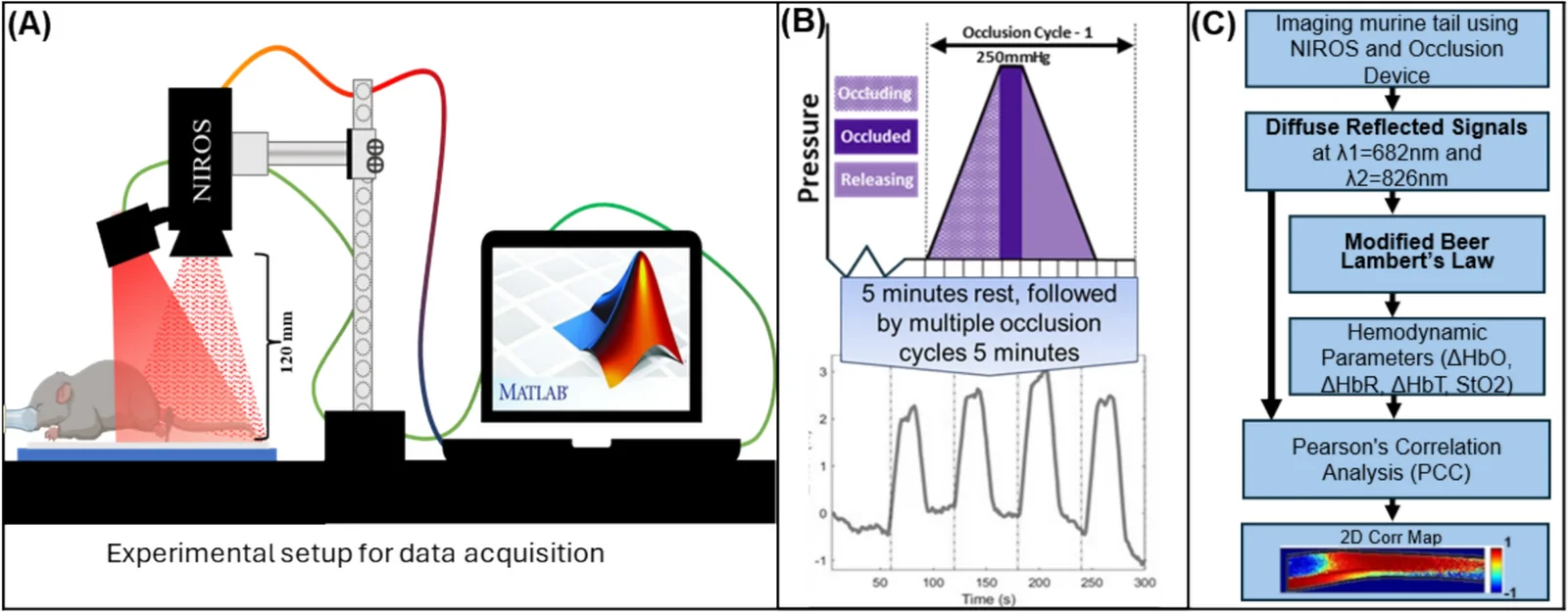
Experimental setup **A** NIROS is placed perpendicular to the imaging plane at 120 mm [8]. Diffuse reflectance signals were obtained from the murine tail using NIROS’s automated data acquisition via a custom-developed MATLAB GUI (graphical user interface). **B** The occlusion paradigm employed during imaging studies and a sample plot of hemodynamic changes across time in response to multiple occlusion cycles. Occlusion was carried out using a commercial device appropriate to occlude the murine tail. **C** Flowchart with stepwise procedure for data analysis. The diffuse reflectance signals at both the NIR wavelengths were used along with the modified Beer-Lambert’s law(mBLL) to obtain the spatiotemporal hemodynamic maps, following which flow correlation using Pearson’s correlation analysis was performed to obtain 2D correlation maps (ranging from − 1 to + 1)

An in-house, non-contact, NIRS-based imaging device (NIROS) was used to image murine tails and obtain the diffuse reflectance NIR signals (shown in Fig. 1A). NIROS utilizes LED-based source light (at 682 nm and 826 nm wavelengths), which is multiplexed at 2 Hz (1 Hz for each wavelength) to acquire the spatiotemporal maps of the effective diffuse reflectance in the murine tail [9]. This portable, continuous wave-based imaging device uses an NIR-sensitive CMOS camera to capture the diffuse reflectance NIR signals from the surface of the murine tail [9, 10]. A MATLAB-based GUI controls NIROS for data acquisition and processing. The experimental setup during non-contact mice imaging is shown in Fig. 1A. The changes in diffuse reflectance in response to a pressure occlusion stimulus were acquired spatiotemporally during the study. The occlusion stimulus was induced using a commercial small animal-compatible pressure occlusion device (CODA monitor, KENT Scientific) (Fig. 1B).

#### Image Acquisition

In this study, the existing raw diffuse reflectance data from our earlier analysis were used and reanalyzed using different image analysis methodologies outlined in the section below. A total of 11 adult mice (CKD = 5, CKD+VC = 6) were used. NIROS was fixed on the table at 12 cm from the imaging plane [8]. Mice were anesthetized in an anesthesia chamber using 1-2% isoflurane and 1–2% oxygen and placed in a dorsal position on a heating mat heated at 35 °C to maintain the mouse’s body temperature [9]. The occlusion cuff from a commercial pressure occlusion device (CODA monitor, Kent Scientific) was attached to the proximal end of the tail to induce complete tail occlusion during imaging. The mice were under anesthesia during imaging, and the anesthesia was controlled based on their visual breathing rate. NIROS was used to image the murine tail during the occlusion. The occlusion paradigm included 5 min of rest followed by multiple occlusion cycles. Each occlusion cycle included 5–6 s to reach the maximum pressure of 250 mmHg, 5 s of hold, 15 s to release the pressure, and 30 s of rest in between the consecutive cycles (Fig. 1B). Imaging was performed on both the CKD and CKD+VC groups in week 6 and week 12 to obtain the diffuse reflectance signals in response to the occlusion paradigm. The diffuse reflectance signals obtained from only the first occlusion cycle were included in the flow pattern correlation analysis.

#### Analysis 1: Correlation Analysis Based on Hemoglobin Parameters

In this analysis, we aim to determine potential changes in hemodynamic flow patterns throughout the tail and correlate these changes to the presence of VC in the aortae.

##### Image Analysis

The occlusion-induced changes in the hemoglobin-based parameters (in terms of oxy-HbO, de-oxy-HbR, total hemoglobin (HbT), and oxygen saturation (StO_2_)) were obtained spatiotemporally using the NIR diffuse reflectance signals acquired at both the NIR wavelengths of 682 and 826 nm (throughout the mouse tail). The modified Beer-Lambert’s law (mBLL) was used to obtain the spatiotemporal maps of effective hemoglobin-based parameters [11–13]. Initially, the changes in optical density (OD_(x,y,t)_) at each wavelength (λ), 2D spatial location (*x*, *y*), and time point (t) were calculated (shown in Eq. 1) using the diffuse reflectance signals obtained at each wavelength (${I}_{({x},{y},\mathrm{t})}^{{\lambda i}})$. The dark noise (${I}_{\mathrm{D}}({x},{y})$) was also considered for each pixel intensity value (${I}_{({x},{y},\mathrm{t})}^{{\lambda i}})$ and a calibration image (${I}_{\mathrm{cal}({x},{y},\mathrm{t})}^{{\lambda i}})$ of a diffusing calibration sheet was acquired at the beginning of each imaging session as a reference image.

1 $$\Delta {\mathrm{OD}}_{{\left( {x,y,t} \right)}}^{\lambda } = - \log \frac{{I_{{\left( {x,y,t} \right)}}^{\lambda i} - I_{{\mathrm{D}}} \left( {x,y} \right)}}{{I_{{{\mathrm{cal}}\left( {x,y,t} \right)}}^{\lambda i} - I_{{\mathrm{D}}} \left( {x,y} \right)}}$$

These optical densities, along with the extinction coefficients for oxyhemoglobin (${\varepsilon }_{\text{HbO }}^{\lambda 1}\mathrm{and} {\varepsilon }_{\text{HbO }}^{\lambda 2})$ and deoxyhemoglobin ${(\varepsilon }_{\text{HbR }}^{\lambda 1}{\mathrm{and}}\varepsilon_{\text{HbR }}^{\lambda 2}$) at each wavelength ($\lambda 1{\mathrm{and}} \lambda 2)$, path length factor B, and inter-optode distance (L), were in turn used to obtain the spatiotemporal maps of HbO and HbR:

2 $${\mathrm{HbO}}\left( {x,y,t} \right) = \frac{{\varepsilon_{{{\mathrm{HbR}} }}^{\lambda 1} \Delta {\mathrm{OD}}^{\lambda 2} - \varepsilon_{{{\mathrm{HbR}} }}^{\lambda 2} \Delta {\mathrm{OD}}^{\lambda 1} }}{{\left( {\varepsilon_{{{\mathrm{HbR}}}}^{\lambda 1} \varepsilon_{{{\mathrm{HbO}} }}^{\lambda 2} - \varepsilon_{{{\mathrm{HbR}}}}^{\lambda 2} \varepsilon_{{{\mathrm{HbO}}}}^{\lambda 1} } \right){\mathrm{LB}}}}$$

3 $${\mathrm{HbR}}\left( {x,y,t} \right) = \frac{{\varepsilon_{{{\mathrm{HbO}}}}^{\lambda 2} \Delta {\mathrm{OD}}^{\lambda 1} - \varepsilon_{{{\mathrm{HbO}} }}^{\lambda 1} \Delta {\mathrm{OD}}^{\lambda 2} }}{{\left( {\varepsilon_{{{\mathrm{HbR}}}}^{\lambda 1} \varepsilon_{{{\mathrm{HbO}}}}^{\lambda 2} - \varepsilon_{{{\mathrm{HbR}}}}^{\lambda 2} \varepsilon_{{{\mathrm{HbO}}}}^{\lambda 1} } \right){\mathrm{LB}}}}$$

where B and L are assumed to be constant at wavelength and, therefore, are grouped to obtain the effective changes in HbO and HbR [9, 10].

4 $$\Delta {\mathrm{HbO}}\left(x,y,t\right)=\left[\mathrm{HbO}\right]\left(x,y,t\right)\mathrm{LB}$$

5 $$\Delta {\mathrm{HbR}}(x,y,t)=[\mathrm{HbR}](x,y,t)\mathrm{LB}$$

The total hemoglobin and oxygen saturation were calculated as

6 $$\Delta {\mathrm{HbT}}(x,y,t)=\Delta {\mathrm{HbO}}\left(x,y,t\right)+\Delta {\mathrm{HbR}}(x,y,t)$$

7 $$\Delta {\mathrm{St}}{\mathrm{O}}_{2}(x,y,t)=[\Delta {\mathrm{HbO}}(x,y,t)]/[\Delta {\mathrm{HbT}}\left(x,y,t\right)]$$

The spatiotemporal changes in the effective hemodynamic parameters were determined in response to an occlusion stimulus in the murine tail. The entire tail region was manually selected and segmented to remove the background and the cuff region. A Savitzky–Golay filter was applied to smoothen each pixel’s time-varying hemodynamic signal and reduce noise [9]. The hemodynamic parameter maps were further initialized with respect to the first time point of the occlusion cycle (i.e., after the 60 s of rest period) before correlation analysis.

##### Pearson’s Correlation Coefficient (PCC) Analysis

The initialized hemodynamic parameter maps (in terms of ∆HbO, ∆HbR, ∆HbT, and StO_2_) of the first occlusion cycle were used to correlate the flow pattern changes in the tail across mouse groups and weeks of imaging, using the median-based Pearson’s correlation coefficient (PCC) analysis. The overall median in the hemodynamic flow pattern was obtained for the respective hemodynamic parameters. The temporal variations in these parameters at each spatial location were compared to the median using PCC analysis (shown in Eq. 8) to obtain the hemodynamic flow correlation maps for each hemodynamic parameter.

8 $${\mathrm{PCC}}(x,y) = \frac{{\sum _{{i = 1}}^{n} (X_{i} - x_{{{\mathrm{md}}}} )(Y_{i} - y_{{{\mathrm{md}}}} )}}{{\sqrt {\sum _{{i = 1}}^{n} (X_{i} - x_{{{\mathrm{md}}}} )^{2} {\mkern 1mu} (Y_{i} - y_{{{\mathrm{md}}}} )^{2} } }}$$

Here, PCC(*x*, *y*) is the Pearson’s correlation coefficient at every imaged pixel (*x*, *y*); *X*_*i*_ and *Y*_i_, respectively, are the respective hemoglobin-based parameters’ value in the *x*^th^ pixel at a given time point, and the value in the *y*^th^ pixel at a given time point respectively; and ${{x}}{\mathrm{md}}$ and ${{y}}{\mathrm{md}}$_,_ are the median value of each hemodynamic parameter at a given wavelength. The output of the 2D Pearson’s correlation map ranges from − 1 to + 1 (i.e., negatively to positively correlated). The obtained correlation maps were in turn used to assess the extent of the overall synchrony or asynchrony in the hemodynamic flow pattern across the tail.

##### Statistical Analysis

Once the correlation maps for each hemodynamic parameter were obtained, five 5 × 10-pixel ROI (250 pixels in total) were randomly chosen by the operator. These regions were chosen from the upper half of the 2D PCC maps of the tail, where noticeable changes in flow pattern were visually observed in week 12 compared to week 6. The average pixel intensity in the selected region of interest from the 2D correlation maps was calculated. These average pixel values (mean PCC values) of the respective hemodynamic flow correlation maps were compared between week 6 and week 12 of each mouse group (CKD and CKD+VC) to assess temporal localization patterns. Paired *t* tests were performed to assess the differences in correlation maps of hemodynamic parameters across the two groups and the weeks (Fig. 4A, B).

#### Analysis 2: Correlation Analysis Based on Diffuse Reflectance

In analysis 1, the NIR diffuse reflectance signals measured at 682 nm (NIR1) and 826 nm (NIR2) were used to calculate the hemodynamic maps using mBLL and determine the flow pattern differences in the tail in the mouse group with and without CKD-induced VC. This mBLL-based hemodynamic analysis assumes a constant path length factor, omits the scattering properties, and assumes a fixed inter-optode distance [16]. Therefore, to avoid these assumptions and keep the data processing simpler, this analysis focused on using the raw NIR diffuse reflectance signals directly to capture the flow pattern differences due to the presence of CKD-induced VC.

##### Image Analysis

The occlusion-induced changes in the diffuse reflectance NIR signals (at both NIR wavelengths: NIR1 and NIR2) were dynamically acquired across the murine tail. Further image analysis was performed following a similar approach to analysis 1; however, instead of hemodynamic parameters, raw diffuse reflectance signals were directly used, followed by tail segmentation and a Savitzky–Golay filter for smoothening the time-varying diffuse reflectance signal. The raw diffuse reflectance signals were initialized using the 60 s time point prior to occlusion, similar to analysis 1.

##### Pearson’s Correlation Coefficient (PCC) Analysis

For PCC analysis, the overall median NIR diffuse reflectance maps from the tail were used and compared with each spatial location across time using Eq. 8. Here, PCC (x, y) denotes the median Pearson’s correlation coefficient at each pixel (*x*, *y*). *X*_i_ and *Y*_i_, respectively, represent the NIRS diffuse reflectance values (at a given NIR wavelength) in the *x*^th^ pixel at a given time point and the value in the *y*^th^ pixel at a given time point, and *x*_md_ and *y*_md_ are the median values of diffuse reflectance signals of the entire tail at their respective wavelength. The median of the PCC (*x*, *y*) distribution was used to evaluate the overall extent of correlation in the NIRS diffuse reflectance-based flow at both wavelengths (NIR1 and NIR2) across the imaged area. The PCC correlation maps were compared between the CKD group (control group, without VC) and the CKD-induced VC group (test group, with VC).

##### Statistical Analysis

The statistical analysis was performed using paired *t* tests similar to analysis 1. The differences in the mean pixel values in the selected region of interest from the 2D maps of NIR1 and NIR2 were compared between week 6 and week 12 on CKD and CKD+VC mouse groups to observe the potential changes in the flow pattern with the presence of VC.

## Results

The Pearson-based correlation maps for the hemodynamic parameters (∆HbO, ΔHbR, ∆HbT, and StO_2_) and NIR-based diffuse reflectance signals (NIR1 and NIR2) were analyzed and compared between the two mouse groups across weeks 6 and 12 for the first occlusion cycle.

### Analysis 1: Correlation Analysis Based on Hemoglobin Parameters

**Fig. 2:**
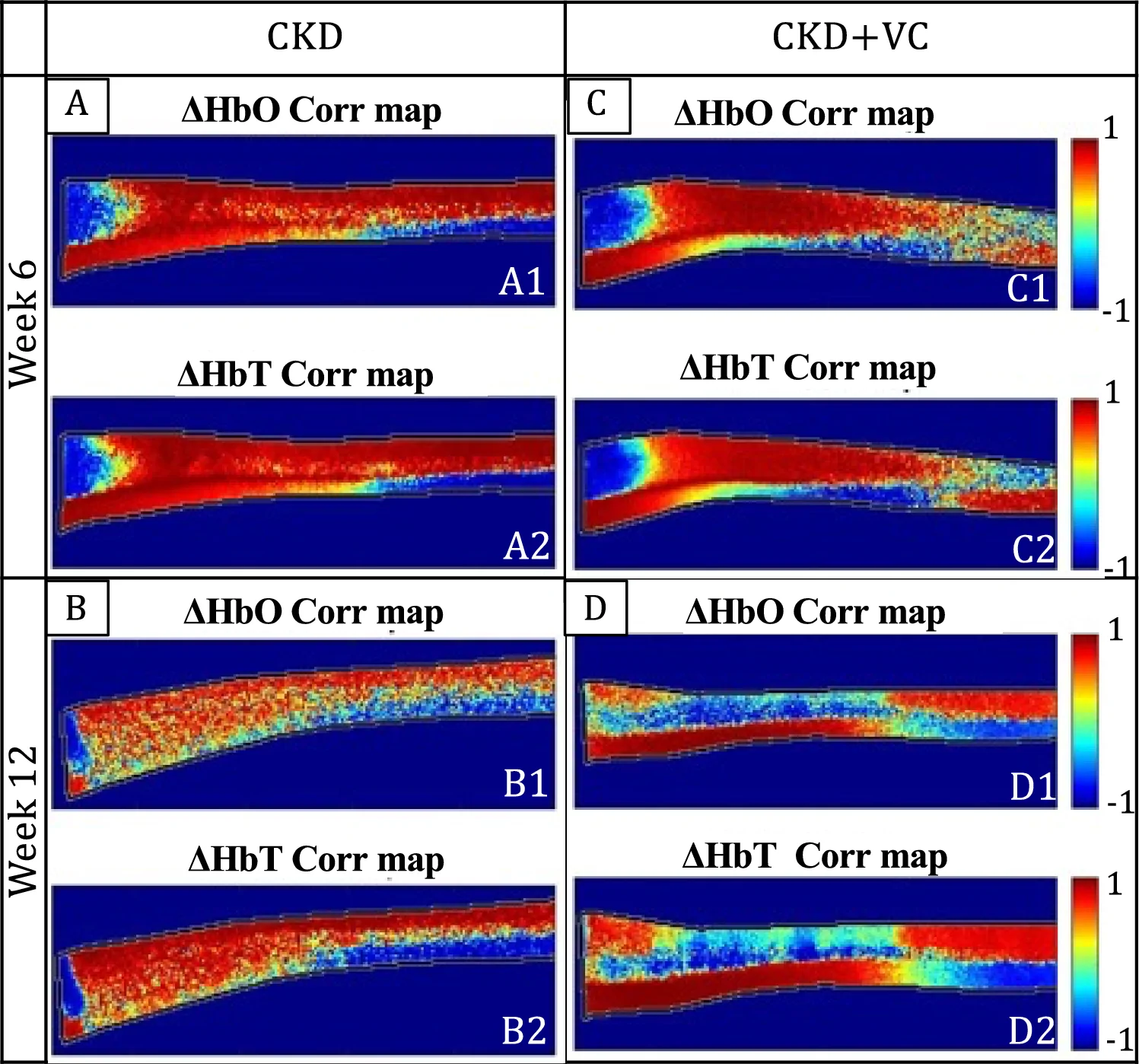
Hemodynamic flow correlation maps based on ∆HbO and ∆HbT, obtained from murine tail, between weeks 6 and 12 across the two mouse groups (CKD and CKD+VC). The correlation values range from − 1 to + 1, where − 1 and + 1 imply negatively and positively correlated, respectively

The PCC maps of ∆HbO and ∆HbT during the first occlusion cycle were compared across weeks 6 and 12 of the two mouse groups qualitatively (shown in Fig. 2) to observe any differences in the flow pattern. There were no distinct flow pattern differences in the correlation map for ΔHbR and StO_2_ (not shown here for brevity). In the CKD group, the ∆HbO and ∆HbT-based hemodynamic correlation maps demonstrated consistently predominant positive correlation at week 12 compared to week 6 (*n* = 4 of 6 mice) (sample case shown in Fig. 2). Subtle variations in flow patterns were also observed in the remaining two mice (*n* = 2 of 6 mice). In contrast, in the CKD+VC group, most mice (*n* = 3 of 5 mice) showed a distinct negative correlation in the ∆HbO-based PCC maps at week 12 compared to week 6 (Fig. 2). The remaining two mice in this group showed increased ∆HbO-based correlation in week 12 compared to week 6. However, these changes were not significant. A similar pattern was observed in the correlation map for total hemoglobin (∆HbT), where *n* = 3 out of 6 mice showed a decrease in ∆HbT-based correlation in week 12 qualitatively (sample case Fig. 2).

**Fig. 3:**
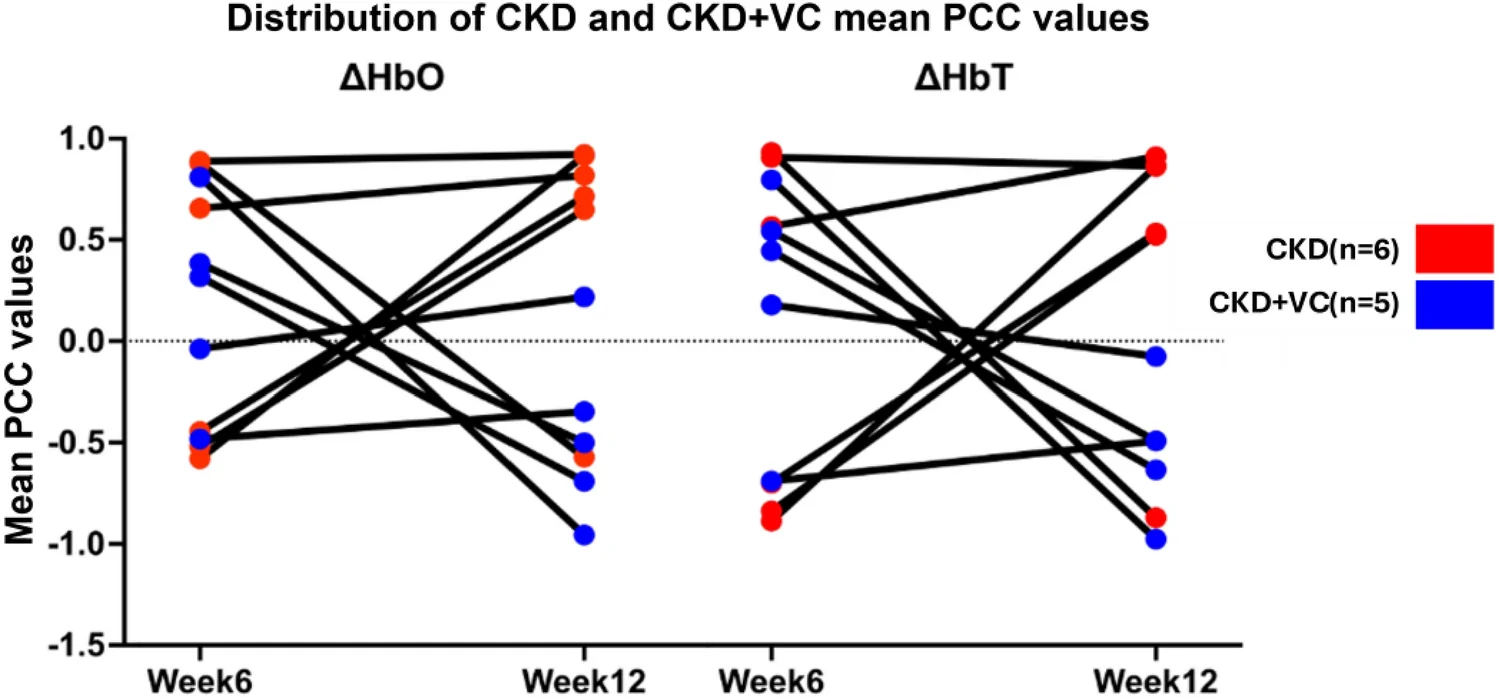
Mean PCC values of randomly selected ROIs from ∆HbO and ∆HbT-based hemodynamic correlation maps for CKD (red dots) and CKD+VC (blue dots) groups at weeks 6 and 12. In most cases, we observed an increase in the PCC values of both ∆HbO and ∆HbT-based correlation maps in the CKD mouse group from week 6 to week 12, and a decrease for both parameters in the CKD+VC mouse group over this same period (from week 6 to week 12)

For statistical analysis, the mean PCC values with all the ROIs for each mouse and each group were compared between week 6 and week 12 (Fig. 3). At week 6, the PCC values did not appear different between CKD and CKD+VC; however, by week 12, we observed differences in these groups. In CKD mice, most cases (*n* = 5 of 6 mice) exhibited an increase in mean PCC values (red dots in Fig. 3 for ∆HbO and ∆HbT, respectively) by week 12. On the contrary, there was a distinct decrease in the mean PCC values in the CKD+VC group (*n* = 4 of 5 mice) in ∆HbO and ∆HbT-based plots.

#### Changes in the CKD and CKD+VC Groups Across Weeks

**Fig. 4:**
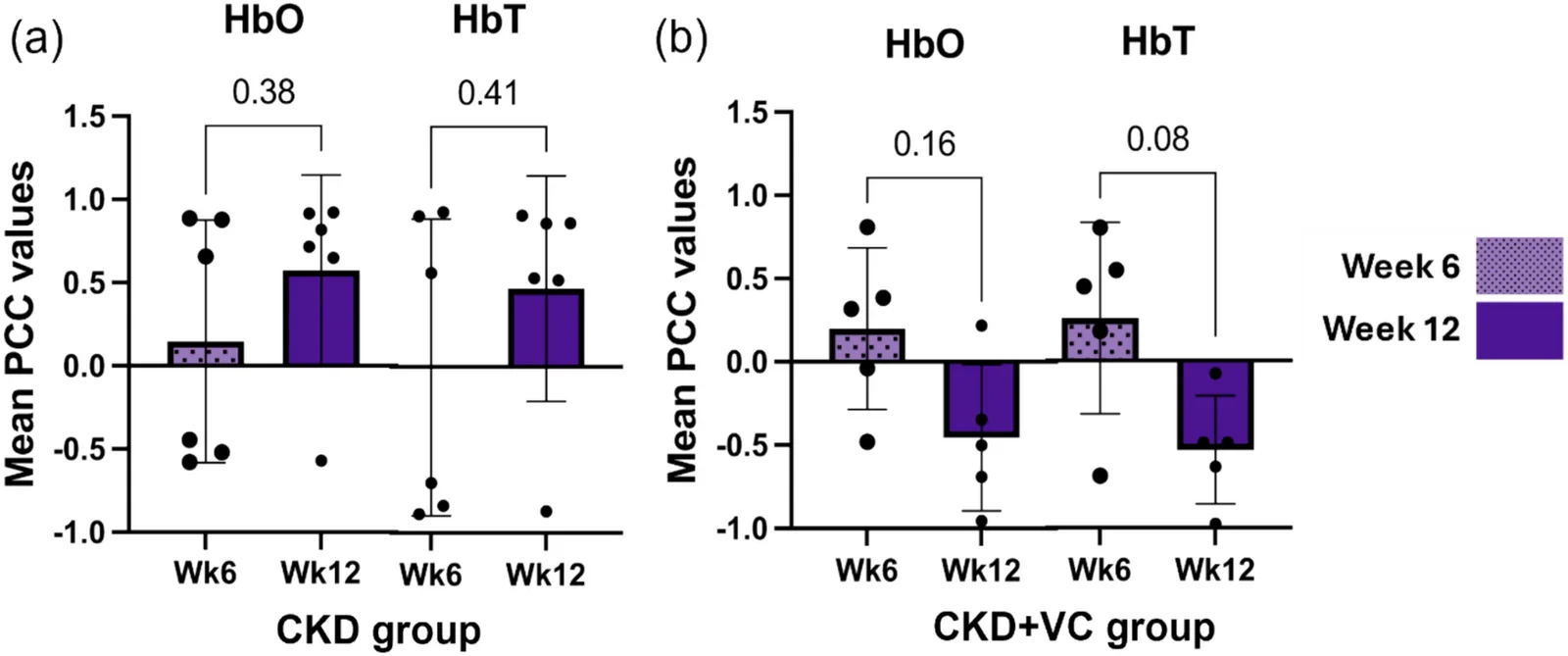
**a** Paired *t* tests were conducted on the mean PCC values of the randomly selected ROI (250 pixels) in the ∆HbO and ∆HbT-based correlation maps for the CKD mouse group, comparing across weeks (6 and 12). The results showed an increase in PCC values, but not statistically significant (*p*-value < 0.05). **b** Paired *t* tests were conducted on the mean PCC values of the randomly selected ROI (250 pixels) in ∆HbO and ∆HbT-based correlation maps for the CKD+VC mouse group, comparing across weeks (weeks 6 and 12). The results showed a significant decrease in PCC values (*p*-value < 0.16) and (*p*-value < 0.08) in ∆HbO and ∆HbT-based correlation maps, respectively.

Figure 4a and b show the results of the paired *t* test across the weeks within each of the CKD and CKD+VC mouse groups, respectively. These results demonstrate that there are no differences between the PCC values in the CKD mouse group across weeks 6 and 12 in both the ∆HbO and ∆HbT-based PCC values (Fig. 4a). On the other hand, only the ∆HbT-based PCC values are statistically different (*p*-value < 0.1) between weeks 6 and 12 of the CKD+VC mouse group (Fig. 4b). The ∆HbO-based PCC values were statistically different at week 12 with *p*-value < 0.2 for this same mouse group.

Upon further analysis of each mouse (*n* = 6 mice), we observed that 4 out of 6 mice showed statistical significance for ∆HbO-based PCC values, and the remaining two were flagged as outliers during statistical analysis. Upon removing these two mice, the paired *t* test across the four mice using ∆HbO-based PCC values also demonstrated statistical significance (*p*-value < 0.1). Since the current study involved a small sample size (*n* = 11 mice), future work will involve a larger sample size of both mouse groups to validate our initial findings.

In summary, hemodynamic flow-based correlation analysis demonstrated that ∆HbT-based flow correlation values can be used to detect the presence of vascular calcification in the CKD+VC mouse group by week 12. No such difference was observed across the weeks in the CKD mouse group that had not developed vascular calcification. In addition, there were no distinct flow pattern differences in the correlation map for ΔHbR and StO_2_ (not shown here for brevity).

### Analysis 2: Correlation Analysis Based on Diffuse Reflectance

**Fig. 5:**
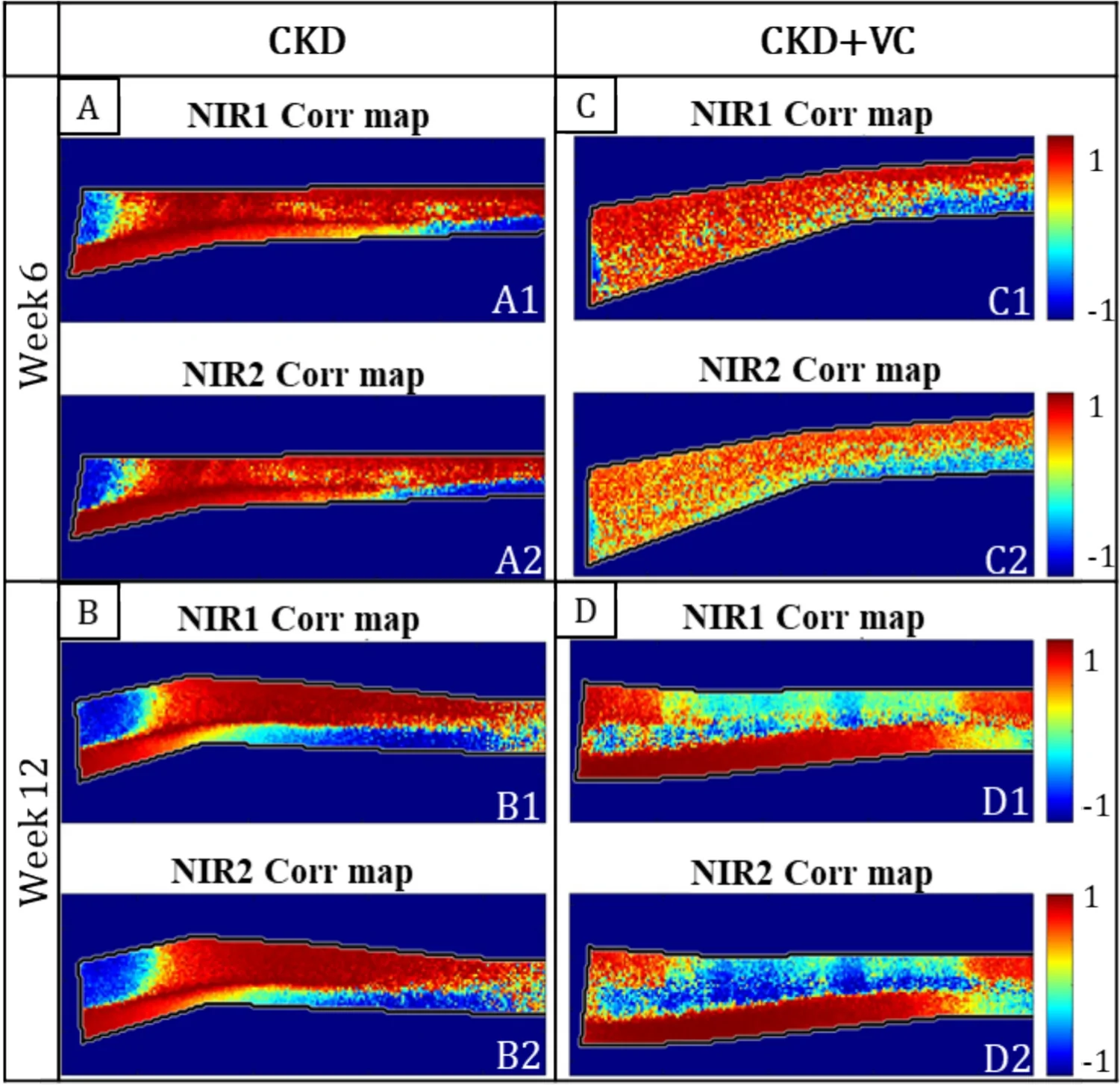
Flow correlation maps based on the diffuse reflectance at NIR1 and NIR2, based on flow correlation maps obtained between weeks (weeks 6 and 12) across the two mouse groups (CKD and CKD+VC). The correlation values range from − 1 to + 1, where − 1 and + 1 imply negatively and positively correlated, respectively

The NIR diffuse reflectance signals from the first occlusion cycle were used to directly obtain the median correlation maps across weeks (weeks 6 and 12) for the two mouse groups (CKD and CKD+VC). The PCC maps based on NIR (NIR1 and NIR2) diffuse reflectance signals followed a pattern similar to the hemodynamic maps showing no changes in the correlation map in the CKD group (Fig. 5A, B) and a distinct decrease in the correlation map in the CKD+VC group by week 12 (shown in Fig. 5C, D). The statistical analysis was performed similar to analysis 1.

**Fig. 6:**
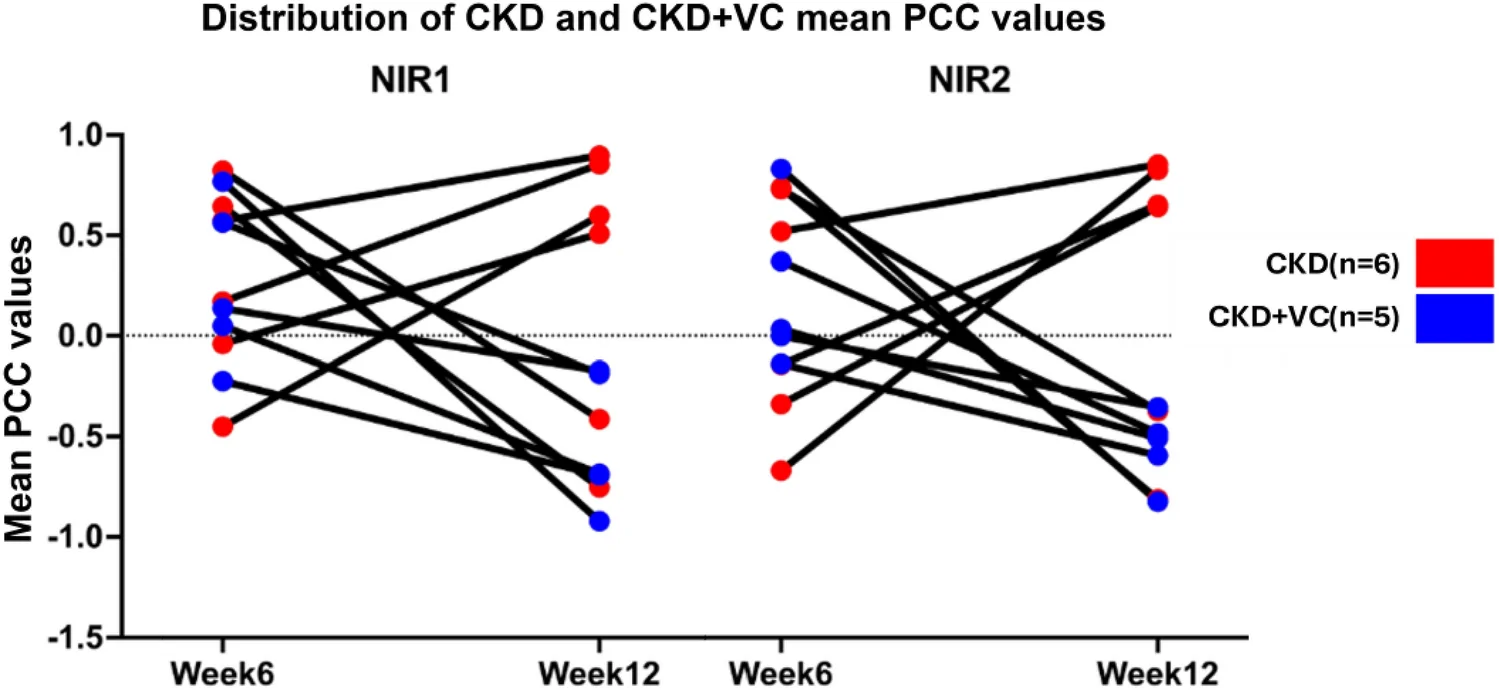
Mean PCC values of randomly selected ROIs from NIR1 and NIR2-based correlation maps for CKD and CKD+VC groups at weeks 6 and 12. Similar to calculated ∆HbO and ∆HbT-based correlation maps data shown in Fig. 3, we observed an increase in the mean PCC values in NIR1 and NIR2 in the CKD mouse group from week 6 to week 12 and a decrease for both parameters in the CKD+VC mouse group over the same period in all mice

Diffuse reflectance-based flow correlation maps based on changes in NIR1 and NIR2 were obtained for both the CKD and CKD+VC groups [14]. The mean PCC values for each mouse and each group were compared between week 6 and week 12 (similar to analysis 1). A similar statistical trend in NIR1 and NIR2 correlation maps was observed where PCC values at week 6 did not appear different between the CKD and CKD+VC groups while at week 12, 4 out of 6 mice in the CKD group showed increases in mean PCC values (red dots in Fig. 6 in NIR1 and NIR2, respectively). However, in the CKD+VC group, there was a distinct drop in the mean PCC values (5 out of 5) in NIR1 and NIR2-based plots (shown in Fig. 6).

#### Changes in the CKD and CKD+VC Groups Across Weeks

**Fig. 7:**
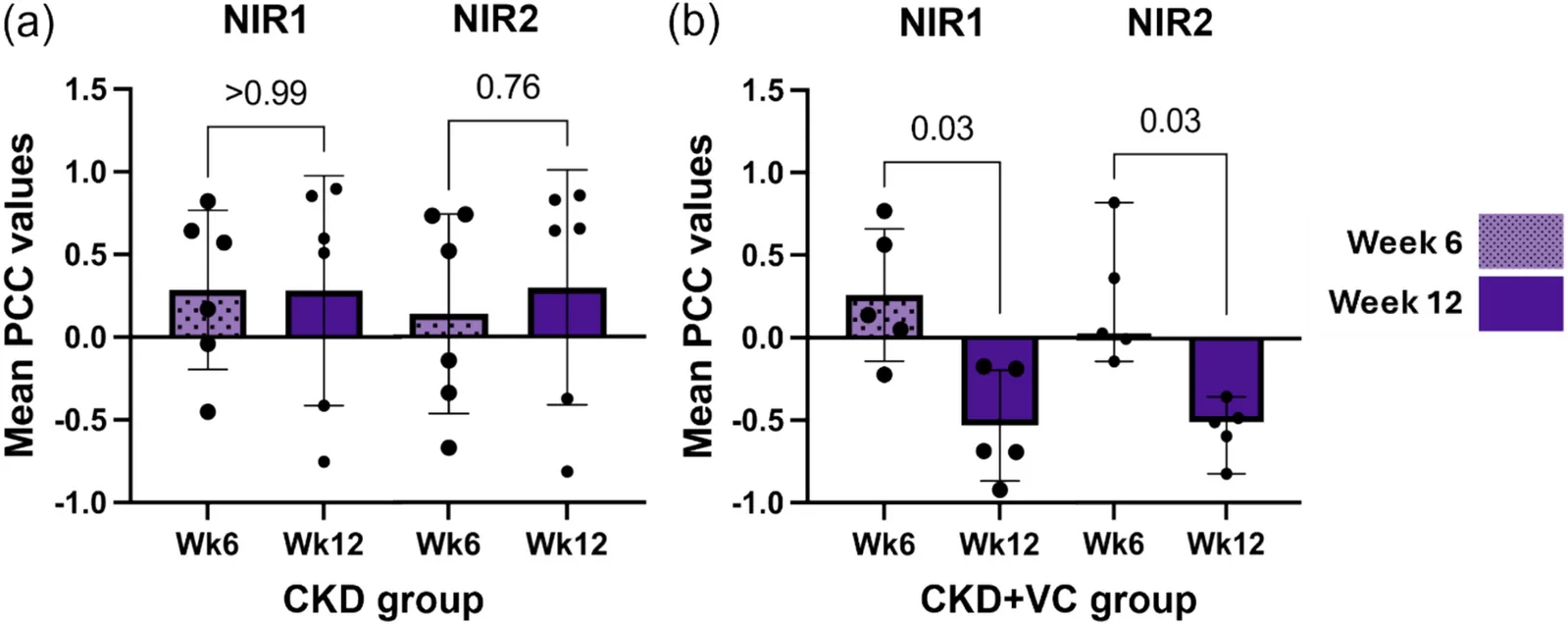
**a** Paired *t* tests were conducted on the mean PCC values of the randomly selected ROI in the NIR1 and NIR2-based PCC maps for the CKD mouse group, comparing across weeks (weeks 6 and 12). No significant differences (*p*-value < 0.05) were observed in either correlation value. **b** Paired *t* tests were conducted on the mean PCC values of the randomly selected ROI in NIR1 and NIR2-based PCC maps for the CKD+VC mouse group, comparing across weeks (weeks 6 and 12). Significant decreases in correlation values (*p*-value < 0.05) were observed in both the NIR1 and NIR2-based PCC values

Figure 7a and b show the results of paired *t* tests across the weeks within each of the CKD and CKD+VC groups, respectively. These results demonstrate that there were no differences between the PCC values in the CKD group across week 6 and week 12 in both NIR1 and NIR2-based PCC values (Fig. 7a). In the CKD+VC mouse group, both the NIR1 and NIR2-based PCC values showed a statistically significant decrease (*p*-value < 0.05) in PCC values at week 12 compared to week 6 (*p*-value < 0.05) (Fig. 7b). Given the small sample in the current study, future work will involve a larger cohort of mice to validate our initial findings. In summary, diffuse reflectance-based flow correlation analysis demonstrated that NIR1 and NIR2 flow correlation values can be used to detect the presence of VC in the CKD+VC mouse group by week 12, and there are no such differences across weeks in the CKD mouse group that had not developed VC.

## Discussion

In this study, mice with CKD and CKD-induced VC were imaged using an NIRS-based imaging approach to assess the spatiotemporal changes in the flow pattern and the extent of its synchrony or asynchrony. At week 12, after the CKD+VC group developed VC, the hemodynamic flow-based parameters (in terms of ΔHbT) shifted predominantly toward a negative correlation, indicating disrupted flow patterns. Studies have shown that CKD mice on an adenine-rich, high-phosphate diet for six weeks develop VC in the aortae [15]. These observed flow pattern changes may be from potential calcification in larger arteries, such as the aortae, possibly leading to arterial stiffness and reduced blood flow in the peripheries. Furthermore, this disruption may also be due to the development of calcification in the peripheral blood vessels [17]. These multifaceted factors, such as vascular geometry, cardiac output, and underlying pathophysiology, may have influenced the observed hemodynamic flow pattern changes and require further investigation. Further studies with a large cohort of both the test (CKD and CKD+VC) and control (healthy) mouse groups are needed. This will help determine whether these changes are solely due to the aortic VC affecting peripheral blood flow or the presence of CKD-induced peripheral calcification.

In addition, this study explored whether diffuse reflectance signals can detect the flow pattern differences in mice with and without VC, similar to hemodynamic correlation maps. This would help to minimize the assumptions made during image processing using the mBLL. The PCC maps derived from diffuse reflectance NIR signals showed negative trends in mice with VC, similar to hemodynamic correlation maps. These trends are likely to be due to changes in blood flow and hemoglobin concentrations, which affect how light is absorbed and reflected within the tissue, resulting in variations in the diffuse reflectance signals. This suggests that diffuse reflectance signals can be used directly to detect the flow pattern differences in mice with and without VC, without the need for hemodynamic flow correlation analysis.

Previous studies have focused on the extent and rate of change at specific points in the tails of mice with CKD and CKD-induced VC [8]. However, these changes could be influenced by the point location selection. This study has overcome these limitations by analyzing the flow pattern differences based on the overall median flow across the entire tail, which can be a potentially simpler indicator for VC detection. This NIRS-based imaging approach has the potential to be a non-contact, quick screening technique for VC detection via peripheral imaging. Nevertheless, it is important to note that this analysis is based on a small sample size (*n* = 11 mice). Ongoing work will focus on observing the flow pattern changes in a large cohort of the test (CKD and CKD+VC) and control (healthy) mouse groups.

Vascular calcification (VC) is the leading cause of mortality in patients with CKD. There is a need for an affordable, accessible technique for longitudinal monitoring of the onset and progression of CKD and VC-related comorbidities [18]. Additionally, identifying potential early indicators is crucial for the timely detection and management of these conditions [19]. Our study demonstrates that NIRS-based imaging can effectively detect peripheral flow pattern changes in mice in the presence of CKD and CKD-induced VC in mice. In the first study, the hemoglobin-based tissue oxygenation parameters were obtained using an in-house NIR optical imaging device (NIROS) and mBLL. The hemodynamic correlation maps were determined in CKD+VC and CKD mice. These correlation maps were distinctly different in the mice between the two groups at week 12, as VC developed. Additionally, we showed that diffuse reflectance alone was sufficient to capture significant changes in peripheral blood flow patterns in VC mice. This approach eliminates the need for mBLL analysis to perform hemodynamic analysis and its related flow correlations.

Therefore, these findings highlight the potential of NIRS-based imaging using diffuse reflectance signals as a quick, low-cost screening technique for assessing and monitoring VC in large arteries by imaging the peripheries. Furthermore, both hemoglobin-based oxygenation correlation maps obtained using mBLL and flow correlation maps derived from raw diffuse reflected signals directly demonstrated the sensitivity to VC-induced changes. Our ongoing work focuses on optimizing the occlusion paradigm to achieve controllable occlusion parameters, allowing for venous, arterial, or total occlusion in the murine tail. This ensures minimal distress to the mice and maximizes the signal contrast between the presence and absence of VC, as well as predicts the onset of VC. Future studies will include validation of these findings in a larger cohort to further establish the reliability and translational potential of the proposed NIRS-based imaging methods.
